# Neoadjuvant immunotherapy plus chemotherapy for resectable non-small cell lung cancer with driver mutations: a retrospective analysis

**DOI:** 10.3389/fimmu.2025.1637615

**Published:** 2025-08-27

**Authors:** Junhao Xu, Jinquan Yao, Yuxin Geng, Jie Huang, Bingwen Zou, Xiao Sun, Jinming Yu, Feifei Teng

**Affiliations:** ^1^ Department of Radiation Oncology, Shandong Cancer Hospital and Institute, Shandong First Medical University and Shandong Academy of Medical Sciences, Jinan, China; ^2^ Department of Outpatient Chemotherapy, Harbin Medical University Cancer Hospital, Harbin, China; ^3^ Department of Radiation Oncology, Lianyungang Clinical College of Nanjing Medical University, Lianyungang, China; ^4^ Department of Radiation Oncology, West China Hospital of Sichuan University, Sichuan, China

**Keywords:** driver gene mutation, immune checkpoint inhibitors, immunotherapy, neoadjuvant, non-small cell lung cancer

## Abstract

**Background:**

Neoadjuvant immunotherapy combined with chemotherapy offers significant benefits for patients with resectable non-small cell lung cancer (NSCLC). However, its efficacy and safety in patients harboring driver gene mutations remain unclear. This study aimed to assess the real-world efficacy and safety of neoadjuvant immunotherapy plus chemotherapy in resectable NSCLC with and without driver gene mutations.

**Methods:**

We retrospectively analyzed patients with NSCLC who received neoadjuvant immunotherapy plus chemotherapy followed by surgical resection. Efficacy was evaluated based on the best radiological response (BRR), major pathologic response (MPR), and pathological complete response (pCR). Survival outcomes were assessed using event-free survival (EFS), and safety was evaluated in all patients.

**Results:**

The study included 73 patients, comprising 34 with driver gene mutations and 39 without driver gene mutations. During the neoadjuvant therapy phase, the BRR rate was 58.8% in the mutated group and 66.7% in the wild-type group (*p* = 0.489). The MPR rate was 47.1% in the mutated group and 41.0% in the wild-type group (*p* = 0.604). The pCR rates were 32.4% and 33.3%, respectively (*p* = 0.929). No significant differences were observed in EFS between the mutated and wild-type groups (*p* = 0.83). Grade 3 treatment-related adverse events occurred in 11.8% of patients with driver gene mutations and 17.9% of patients without driver gene mutations; no Grade 4 or 5 adverse events were reported.

**Conclusion:**

Neoadjuvant immunotherapy plus chemotherapy remains a promising treatment option for patients with resectable NSCLC, irrespective of genetic mutation status.

## 1 Introduction

Therapies involving immune checkpoint inhibitors (ICIs) targeting programmed cell death protein 1 (PD-1) and programmed death ligand 1 (PD-L1) have demonstrated significant improvements in the overall survival (OS) of patients with advanced non-small cell lung cancer (NSCLC) (1–4). However, patients with driver gene mutations have traditionally derived limited clinical benefit from ICIs (5–7). A retrospective study evaluating the efficacy of ICIs in advanced NSCLC patients with driver gene mutations reported a partial best response rate of 19%. Furthermore, the median progression-free survival was 2.8 months, and the median OS reached 13.3 months (5). The disappointing outcomes observed in previous trials have led to the exclusion of patients with driver gene mutations in most registered trials (8–13).

ICIs have shown promise as neoadjuvant therapies in resectable NSCLC. However, the exclusion of patients with driver gene mutations from most clinical trials has resulted in limited clinical evidence regarding their efficacy in this subgroup (8, 9, 13, 14). Based on limited clinical trial data, we observed the unexpected benefits of ICIs in patients harboring gene mutations in resectable NSCLC, which contradicts the findings in advanced NSCLC (15–18). In the epidermal growth factor receptor (EGFR) mutated subgroup analysis of the KEYNOTE-091 (16), pembrolizumab exhibited markedly greater efficacy in patients with EGFR-mutated tumors (hazard ratio [HR]: 0.44, 95% confidence interval [CI]: 0.23–0.84) than those with EGFR-negative tumors (HR: 0.78, 95% CI: 0.59–1.05) or unknown-status tumors (HR: 0.82, 95% CI: 0.63–1.05). Similarly, in the KEYNOTE-671 trial, subgroup analysis of event-free survival (EFS) showed that pembrolizumab led to a pronounced benefit in patients with EGFR mutations (HR: 0.09; 95% CI: 0.01–0.74) (17). While these findings require cautious interpretation due to limited sample sizes, they suggest that neoadjuvant immunotherapy may confer differential benefits depending on specific driver mutation profiles.

Motivated by these unexpected findings, the present study focused on patients with resectable NSCLC harboring driver gene mutations who received neoadjuvant immunotherapy combined with chemotherapy. This study aimed to characterize the real-world landscape of neoadjuvant immunotherapy plus chemotherapy in resectable NSCLC with driver mutations and to evaluate its clinical efficacy and safety in this specific population.

## 2 Materials and methods

### 2.1 Patients

Between May 2021 and August 2023, we retrospectively enrolled adults with untreated, biopsy-confirmed stage IB–IIIB resectable NSCLC (staged according to the American Joint Committee on Cancer, 8^th^ edition) (19). A multidisciplinary team at Shandong Cancer Hospital and Institute, certified these patients as suitable for neoadjuvant immunotherapy combined with chemotherapy. Patients without genetic testing results were excluded from this study. Based on the genetic testing results, patients were categorized into two groups: those with driver gene mutations and without driver gene mutations. Medical records were reviewed to collect the patients’ baseline characteristics, including sex, age, Eastern Cooperative Oncology Group (ECOG) performance status score, PD-L1 tumor proportion score (TPS) assessed utilizing the PD-L1 IHC22C3 pharmDx assay, smoking status, histological features, and follow-up data. The driver gene mutation status was assessed using specimens obtained from the Department of Pathology at Shandong Cancer Hospital and Institute, with genetic testing performed on post-operative biopsy samples in 62 patients (84.9%) and on pre-treatment specimens in 11 patients (15.1%).

### 2.2 Treatment

In this study, patients were treated with at least one cycle of immunotherapy-based neoadjuvant therapy plus either albumin-bound paclitaxel and platinum-based chemotherapy (in patients with squamous histological features) or pemetrexed and platinum-based chemotherapy (in those with non-squamous histological features) every 3 weeks pre-surgical resection. Treatment could be discontinued or delayed in cases of intolerable adverse events (AEs). Surgery was performed within 4 weeks of the final dose of neoadjuvant therapy. **Supplementary Table S1** summarizes the neoadjuvant immunotherapy regimens and surgical resection procedures (See **Supplementary Material**).

### 2.3 Assessment

Tumor size changes were evaluated using contrast-enhanced computed tomography (CT) at baseline, during the neoadjuvant treatment phase, and prior to surgery, in accordance with the Response Evaluation Criteria in Solid Tumors, version 1.1 (RECIST 1.1) (20). During the neoadjuvant therapy phase, patients underwent weekly laboratory blood tests. The pathological response was assessed by examining hematoxylin and eosin (HE)-stained slides of the resected primary tumor and lymph nodes. Laboratory abnormalities and treatment-related AEs were documented in the medical records and graded according to the Common Terminology Criteria for Adverse Events, version 4.03.

### 2.4 Outcomes

The primary endpoint of the neoadjuvant therapy phase was the best radiological response (BRR) rate, defined as the proportion of patients achieving either CR or PR in all evaluable local lesions, as assessed radiologically. Primary postoperative endpoints included pathological responses, specifically major pathologic response (MPR) and pathological complete response (pCR). MPR was defined as ≤10% residual viable tumor cells in HE slides from the primary tumor and sampled lymph nodes. pCR was defined as the complete absence of viable tumor cells in the resected lung specimen and associated lymph nodes. Resected samples with >10% residual viable tumor cells were defined as non-MPR. The secondary endpoint was EFS, defined as the time from the initiation of neoadjuvant immunotherapy-based treatment to recurrence at the surgical site, disease progression at any site, or death from any cause.

During the postoperative follow-up period, patients underwent CT scans at least once every 3 months for the first 2 years and every 6 months thereafter.

### 2.5 Statistical analyses

Continuous variables are presented as the median and interquartile range. Categorical variables were compared using the Chi-square test or Fisher’s exact test, as appropriate. EFS was assessed using the Kaplan–Meier method and compared between groups using the log-rank test. Statistical significance was set at *p <*0.05. All analyses were performed using R software version 4.2.2.

## 3 Results

### 3.1 Patients

**Figure 1:**
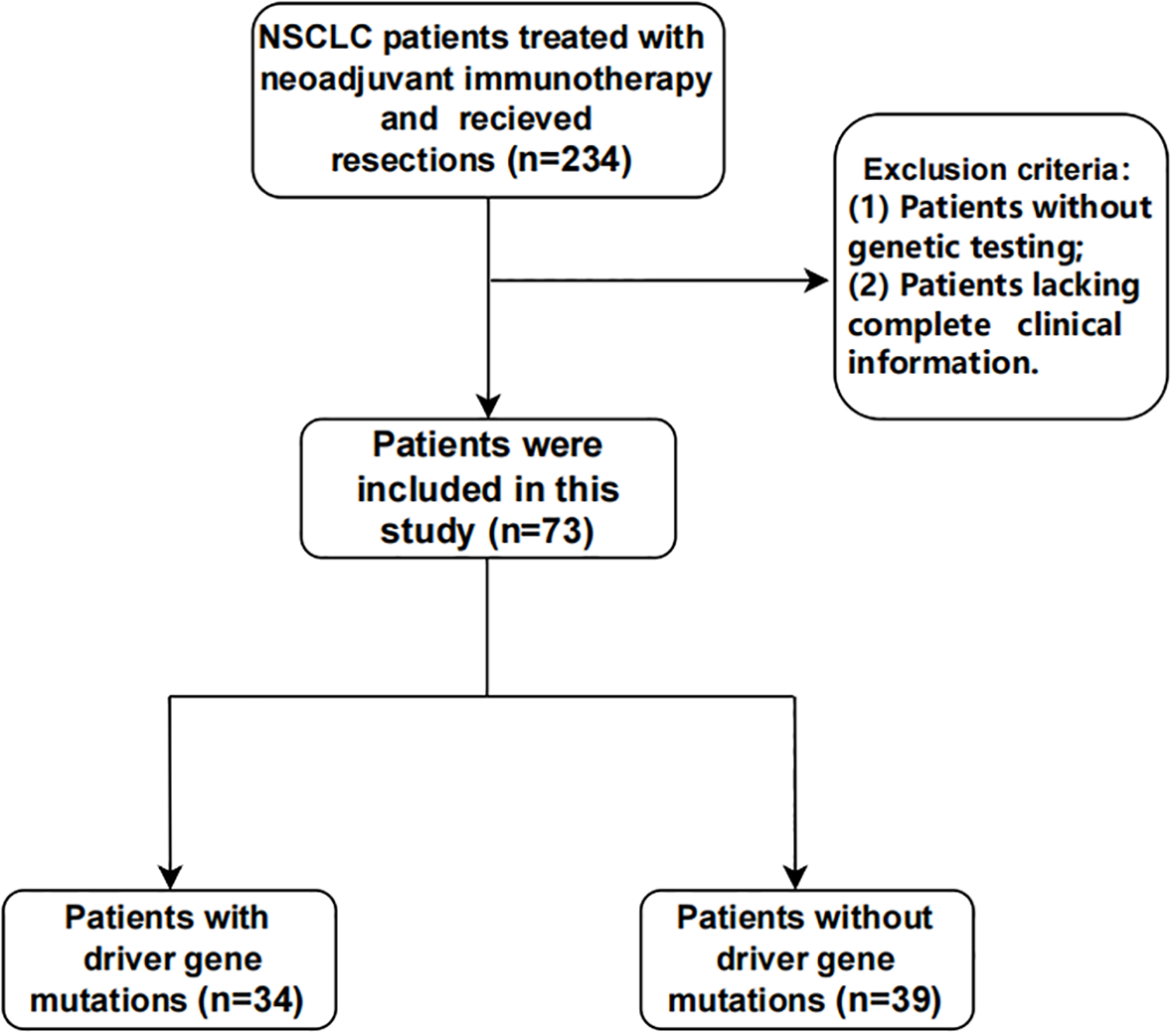
Recruitment and selection process of patients.

Seventy-three patients were retrospectively enrolled between May 2021 and August 2023 (**Figure 1**). Among them, 46.6% (n = 34) harbored driver gene mutations and were assigned to the mutated group, while 53.4% (n = 39) had wild-type tumors and were assigned to the wild-type group.

**Table 1** summarizes the baseline demographic and disease characteristics of the patients. The median age of the patients was 62 years (range, 40–73). PD-L1 tumor proportion score (TPS) was assessed in 63% of patients. Patients with PD-L1 assessment were stratified into three categories based on expression levels: <1%, 1–49%, and ≥50%. Baseline characteristics, such as age, sex, ECOG performance status score, disease stage, tumor stage, nodal stage, and PD-L1 TPS were well-balanced, with exceptions for smoking status and pathological types. A history of smoking was reported in 38.2% (n = 13) of patients in the mutated group, compared with 74.4% (n = 29) in the wild-type group. Histologically, non-squamous features were present in 32 (94.1%) patients in the mutated group compared with only 18 (46.2%) patients in the wild-type group.

**Table 1:** Patient demographics, disease characteristics, and mutations at baseline.

	Mutated (*n* = 34)	Wild-type (*n* = 39)	*p*-values
**Age, years, *n* (%)**			0.3336
<60	16 (47.1)	14 (35.9)	
≥60	18 (52.9)	25 (64.1)	
**Male sex, *n* (%)**	23 (67.6)	33 (84.6)	0.0871
**ECOG performance status score, *n* (%)**			0.2232
0	7 (20.6)	13 (33.3)	
1	27 (79.4)	26 (66.7)	
**Smoking status, *n* (%)**			***0.0018***
Never smoker	21 (61.8)	10 (25.6)	
Former or current smoker	13 (38.2)	29 (74.4)	
**Stage at baseline, *n* (%)** ^a^			0.5978
IB or IIA	1 (2.9)	3 (7.7)	
IIB	6 (17.7)	5 (12.8)	
IIIA	17 (50.0)	23 (59.0)	
IIIB	10 (29.4)	8 (20.5)	
**Tumor stage, *n* (%)**			0.3751
T1b	3 (8.8)	0	
T1c	1 (2.9)	4 (10.3)	
T2a	8 (23.5)	10 (25.6)	
T2b	5 (14.7)	9 (23.1)	
T3	9 (26.5)	9 (23.1)	
T4	8 (23.5)	7 (17.9)	
**Node stage, *n* (%)**			0.8856
N0	6 (17.6)	6 (15.4)	
N1	6 (17.6)	8 (20.5)	
N2	21 (61.8)	25 (64.1)	
N3	1 (2.9)	0	
**PD-L1 tumor proportion score, n (%)**			0.1916
≥50%	6 (17.6)	9 (23.1)	
1–49%	7 (20.6)	12 (30.8)	
<1%	4 (11.8)	8 (20.5)	
Unknown	17 (50.0)	10 (25.6)	
**Histological features, *n* (%)**			***<0.001***
Nonsquamous	32 (94.1)	18 (46.2)	
Squamous	2 (5.9)	21 (53.8)	
**Driver mutation status** ^b^			
KRAS	13 (38.2)	/	
EGFR	11 (32.4)	/	
Her-2	3 (8.8)	/	
MET	2 (5.9)	/	
STK11	2 (5.9)	/	
ROS1	2 (5.9)	/	
ALK	1 (2.9)	/	
BRAF	1 (2.9)	/	
NRAS	1 (2.9)	/	

Bold values indicate statistical significance (p < 0.05).

ECOG, Eastern Cooperative Oncology Group; KRAS, Kirsten rat sarcoma viral oncogene homolog; EGFR, epidermal growth factor receptor; Her-2, human epidermal growth factor receptor 2; MET, cellular-mesenchymal to epithelial transition factor; STK11, serine-threonine kinase 11; ROS1, ROS proto-oncogene 1; ALK, anaplastic lymphoma kinase; BRAF, v-RAF murine sarcoma viral oncogene homolog B1; NRAS, neuroblastoma RAS viral oncogene homolog.

Percentages may not total 100 because of rounding.

a The stage at baseline was evaluated according to the staging criteria of the American Joint Committee on Cancer, 8^th^ edition.

b Some patients have more than one mutation.

The two most common driver gene mutations in the mutated group were the Kirsten rat sarcoma viral oncogene homolog (KRAS; *n* = 13, 38.2%) and EGFR (*n* = 11, 32.4%). Additional mutations included Her-2, MET, STK11, ROS1, ALK, BRAF, and NRAS (**Table 1**).

### 3.2 Efficacy

#### 3.2.1 Radiological outcomes

**Figure 2:**
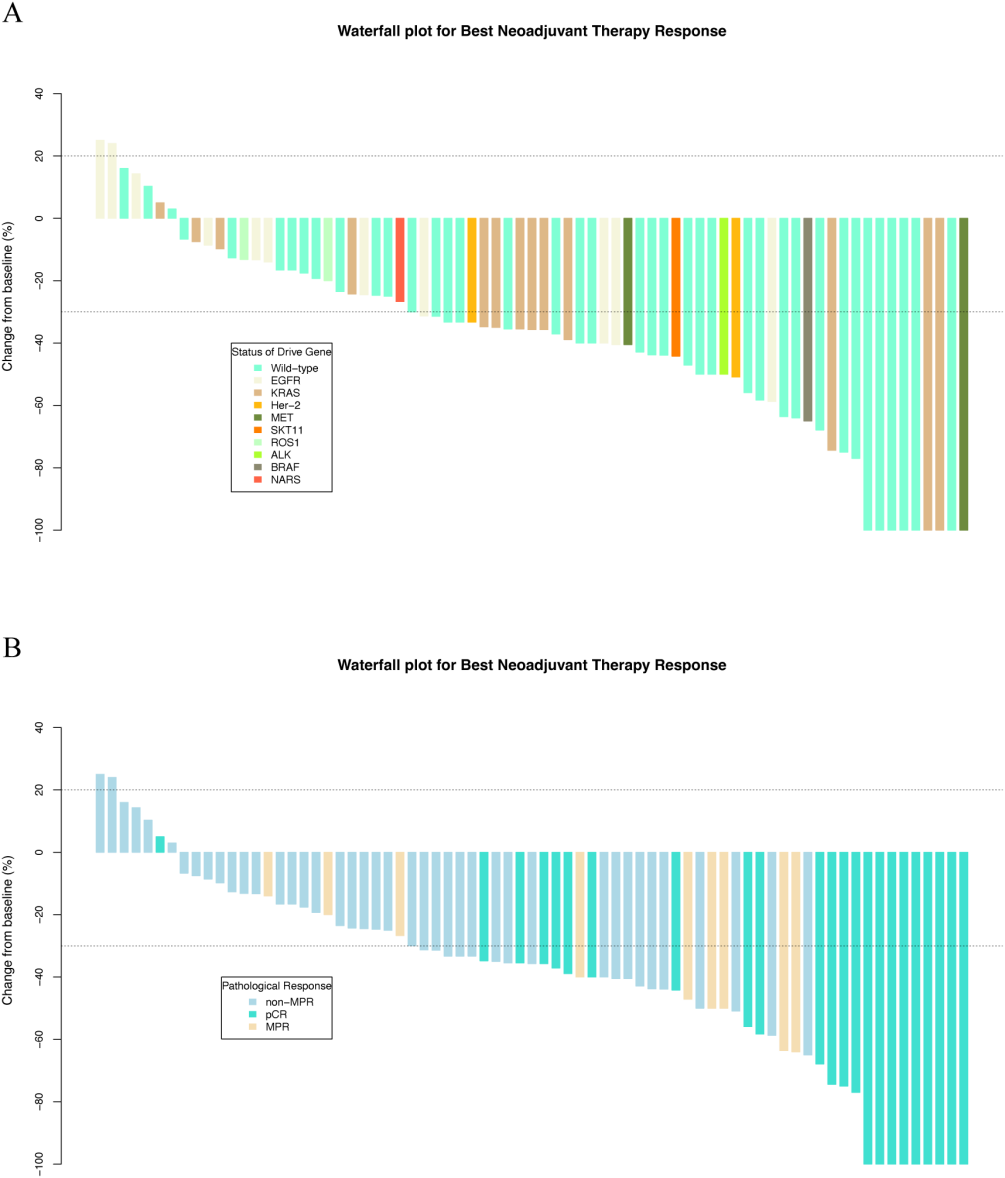
Waterfall plots of best neoadjuvant therapy response stratified by **(A)** driver gene status and **(B)** pathological response.

**Figure 3:**
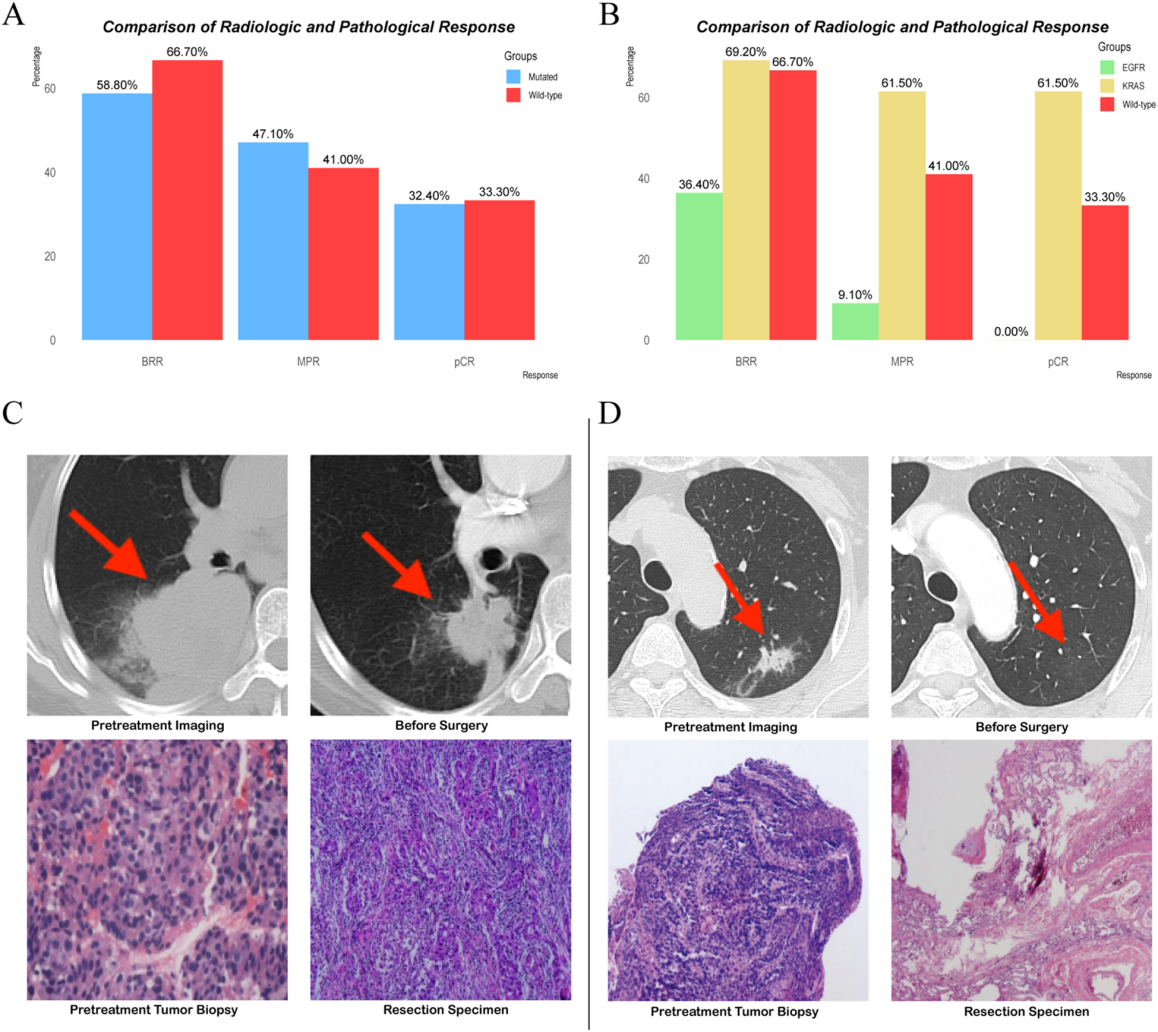
**(A, B)** Comparison of radiologic and pathological response. **(C, D)** Representative radiological and pathological images from patients with EGFR mutations and KRAS mutations.

During the neoadjuvant immunotherapy phase, one patient received only a single cycle of immunotherapy-based treatment, which was discontinued because of a grade 3 AE. All other patients completed two to four treatment cycles, with the specific number of cycles determined by the treating physician based on clinical judgment [**Supplementary Table S1** (See **Supplementary Material**)]. Among the 73 patients, 46 (63.0%) achieved a radiological response, including 9 (12.3%) with CR and 37 (50.7%) with PR. Additionally, 25 (34.2%) patients exhibited stable disease (SD) as their best response, and 2 (2.7%) experienced progressive disease (PD). The BRR rate of all patients was 63.0% (46/73; **Table 2**, **Figures 2A, B**). According to the RECIST 1.1 guidelines, in the mutated group, 20 patients had a radiological response of CR (*n* = 3)/PR (*n* = 17), and 14 patients had SD (*n* = 12)/PD (*n* = 2). In the wild-type group, 26 patients had a radiological response of CR (*n* = 6)/PR (*n* = 20), 13 patients exhibited SD, and no patient experienced PD (**Table 2**, **Figures 2A, B**). The BRR rate for the two groups was 58.8% (95% CI, 40.7–75.4%) in the mutated group and 66.7% (95% CI, 49.8–80.9%; *p* = 0.489) in the wild-type group, no significant differences were found (**Table 2**, **Figure 3A**).

**Table 2:** Outcomes of the best radiological response and pathological response.

	Mutated (*n* = 34)	Wild-type (*n* = 39)	*p*-value
Best radiological response, *n* (%)^a^			
CR	3 (8.8)	6 (15.4)	
PR	17 (50.0)	20 (51.3)	
SD	12 (35.3)	13 (33.3)	
PD	2 (5.9)	0	
BRR (%)	20 (58.8)	26 (66.7)	0.489
Pathological response, *n* (%)			
MPR	16 (47.1)	16 (41.0)	0.604
pCR	11 (32.4)	13 (33.3)	0.929

Data are presented as *n* (%) or percentage.

CR, complete response; PR, partial response; SD, stable disease; PD, progressive disease; BRR, best radiological response rate; pCR, pathological complete response; MPR, major pathological response.

Percentages may not total 100 because of rounding.

a Evaluated by radiological data from baseline to before surgical resection according to the Response Evaluation Criteria in Solid Tumors, version 1.1 (RECIST 1.1).

We further analyzed the BRR rate according to specific driver gene mutations, focusing on the KRAS (*n* = 13) and EGFR (*n* = 11) subgroups. In patients with KRAS mutations, the BRR rate was 69.2% (9/13; 95% CI: 38.6–90.9%), and no cases of PD were observed. Among patients with EGFR mutations, the BRR rate was 36.4% (4/11; 95% CI: 10.9–69.2%), with one patient experiencing PD (**Figure 3B**). When compared with the wild-type group (BRR rate: 66.7%), the KRAS-mutated subgroup showed a similar BRR rate (69.2%), with no significant difference (*p* = 1.000; **Figure 3B**). The BRR rate in the EGFR-mutated subgroup (36.4%) was numerically lower than that in the wild-type group, although the difference did not reach statistical significance (*p* = 0.143; **Figure 3B**).

#### 3.2.2 Pathological outcomes

Lobectomy was the predominant surgical procedure performed during the surgical phase. The complete resection (R0) rates were 89.5% and 97.1% in the two groups [**Supplementary Table S1** (See **Supplementary Material**)]. After surgical resection, 43.8% (32/73) of patients achieved MPR, and 32.9% (24/73) reached pCR. Patients who achieved BRR were more likely to achieve MPR or pCR, demonstrating a correlation between the radiological response and pathological outcomes (**Table 2**, **Figure 2B**).

In the mutated group, the MPR rate was 47.1% (95% CI: 29.8–64.9%) compared to 41.0% (95% CI: 25.6–57.9%) in the wild-type group, with no statistically significant difference (*p* = 0.604) (**Table 2**, **Figure 3A**). Similarly, the pCR rates were 32.4% (95% CI: 19.6–51.4%) in the mutated group and 33.3% (95% CI: 17.4–50.5%) in the wild-type group, also showing no significant difference (**Table 2**, **Figure 3A**).

In the KRAS-mutated subgroup, all individuals who achieved MPR also achieved pCR. This trend was observed in 61.5% of cases (8/13; 95% CI, 31.6–86.1%; **Figure 3B**). Compared with that in the KRAS-mutated subgroup, the MPR rate in the EGFR-mutated subgroup was significantly lower at 9.1% (1/11; 95% CI, 0.2–41.2%; *p* = 0.026; **Figure 3B**). Additionally, none of the patients in the EGFR-mutated subgroup achieved pCR (**Figure 3B**). When compared with the wild-type group, the KRAS-mutated subgroup had a numerically higher MPR rate, although the difference was not statistically significant (*p* = 0.199; **Figure 3B**). The wild-type group had a better MPR rate than the EGFR-mutated subgroup, but this difference also did not reach statistical significance (9.1% vs 41.0%; *p* = 0.2296; **Figure 3B**). The pCR rate in the KRAS-mutated subgroup was 61.5%, compared with 33.3% in the wild-type group (*p* = 0.105). However, the pCR rate in the EGFR-mutated subgroup was significantly lower at 0%, compared with 33.3% in the wild-type group (*p* = 0.046; **Figure 3B**).

Representative radiological and pathological images from these patients were selected to illustrate these findings. After receiving neoadjuvant therapy, the patients with EGFR mutations achieved a BRR of PR and a pathological response of non-MPR (**Figure 3C**). However, in the patients with KRAS mutations, the tumor disappeared radiologically, and the pathological response reached pCR (**Figure 3D**).

#### 3.2.3 Survival analysis

**Figure 4:**
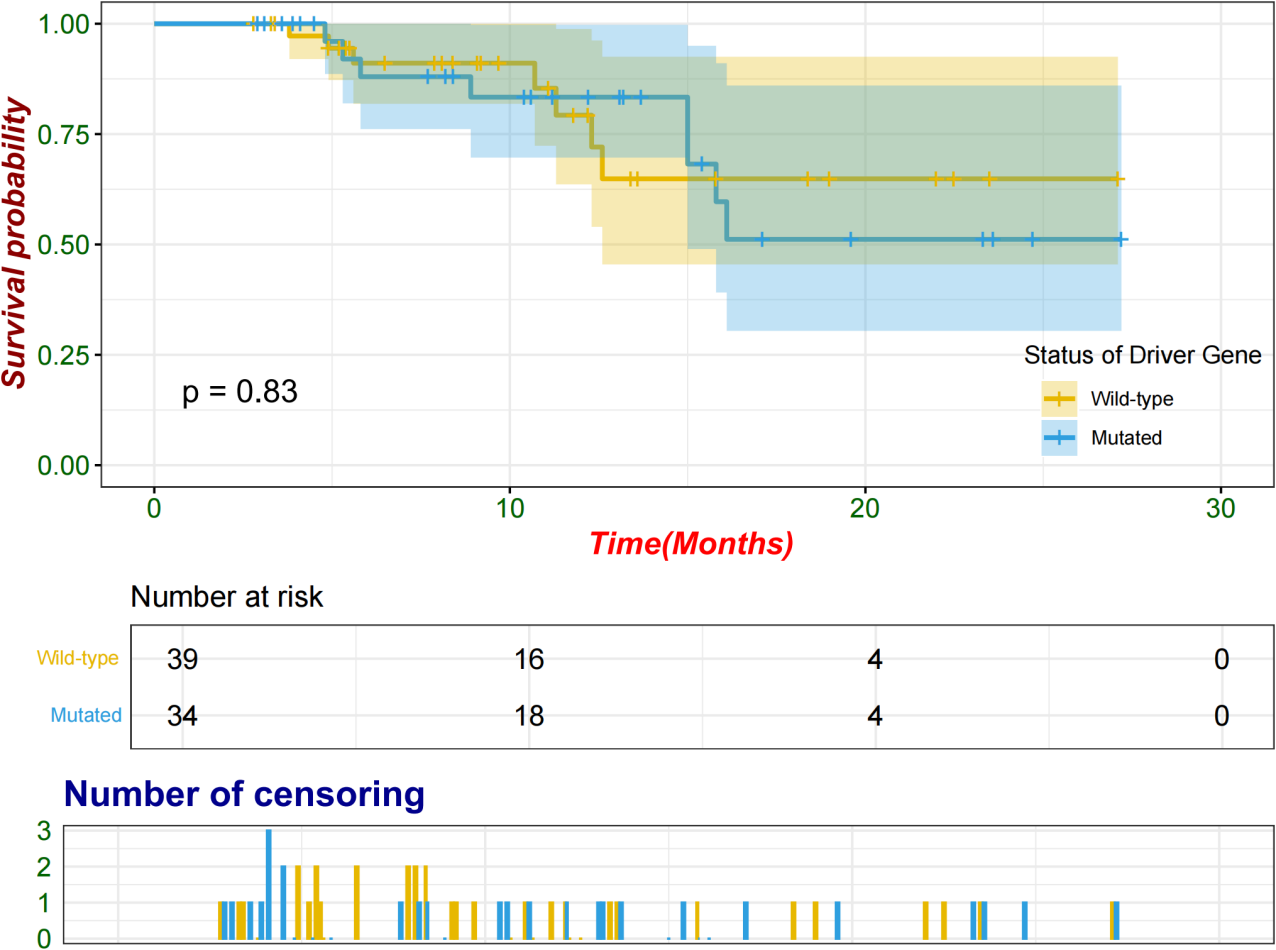
Kaplan–Meier survival curves of all patients.

Following a period extending from the initiation of neoadjuvant therapy to the cutoff date, compared with seven patients (17.9%) in the wild-type group, eight (23.5%) patients in the mutated group experienced relapse either at the surgical site or in other organs. The median follow-up time was 11.1 months. The median EFS was not reached in either group, and no significant difference was observed between the groups (log-rank p = 0.83; **Figure 4**). An exploratory analysis was conducted across various subgroups, including sex, age, ECOG performance status, smoking status, histological features, baseline stage, and PD-L1 TPS, to evaluate potential associations with EFS (**Supplementary Figure S1**). No significant differences in the EFS were observed between the mutated and wild-type groups across the subgroups.

### 3.3 Safety

Treatment-related AEs of any grade were observed in 93.2% (68/73) of patients, with 97.1% (33/34) in the mutated group and 87.2% (34/39) in the wild-type group experiencing such events. The most common AEs were anemia (79.4% in the mutated group vs 64.1% in the wild-type group), nausea (50% vs 38.5%), fatigue (38.2% vs 43.6%), and vomiting (38.2% vs 28.2%). Grade 3 treatment-related AEs occurred in 11.8% (4/34) of patients in the mutated group and 17.9% (7 of 39) of patients in the wild-type group. Only one patient discontinued neoadjuvant therapy due to a grade 3 thrombocytopenia. No grade 4 or 5 AEs were reported, and no new toxicity signals were observed [**Supplementary Table S2** (See **Supplementary Material**)].

## 4 Discussion

CheckMate-816 is the first Phase III study to show that neoadjuvant immunotherapy combinations offer significant clinical benefits for patients with NSCLC (9). However, patients with EGFR mutations were not included in the CheckMate-816 study. In previous subgroup analyses of the large neoadjuvant immunotherapy clinical trial KEYNOTE-671 in NSCLC (17), patients with EGFR mutations exhibited favorable clinical outcomes compared with those with wild-type or unknown EGFR status. Additionally, subgroup analyses from the IMpower010 and KEYNOTE-091 trials indicated that adjuvant ICI provided a DFS benefit in the EGFR mutated-subgroup (15, 16). These findings motivated us to investigate the efficacy and safety of neoadjuvant immunotherapy in patients with resectable NSCLC harboring driver gene mutations. In our study, the mutated group included several heterogeneous driver mutations, and this necessary simplification might obscure mutation-specific effects. Contrary to previous studies that reported limited efficacy of immunotherapy in patients with NSCLC and driver gene mutations (5–7), neoadjuvant immunotherapy demonstrated comparable efficacy in patients with NSCLC and driver gene mutations, showing good safety profiles. No significant differences in radiological and pathological responses or EFS were observed between the mutated and wild-type groups. These findings suggest that neoadjuvant immunotherapy is a viable treatment option for patients with resectable NSCLC, regardless of their genetic mutation status. Adopting this strategy can prevent treatment delays associated with waiting for genetic testing results and mitigate the inaccuracies and economic costs associated with preoperative genetic evaluations using biopsy specimens.

In our study, the BRR rate in the mutated group was significantly higher than the rates reported in previous studies (5, 21). These contradictory results may be attributed to the distinct tumor microenvironments (TME) observed in patients with resectable versus advanced NSCLC (22, 23). Differences in the TME can significantly influence the efficacy of treatment modalities across different disease stages. In patients with resectable NSCLC, the TME is more conducive to activating antitumor immunity. the TME in advanced disease is typically more immunosuppressive, likely due to progressive tumor evolution and chronic immune evasion, thereby diminishing the efficacy of ICIs (24–26).

KRAS is the most prevalent oncogenic alteration in NSCLC, occurring in approximately 30% of adenocarcinoma cases (27). Previous studies have demonstrated that KRAS mutations in NSCLC can activate downstream signaling pathways that promote PD-L1 expression in tumor cells (28, 29). The increased expression of PD-L1 facilitates immune evasion and drives tumor progression. The expression of PD-L1 is significantly higher in KRAS-mutated tumors than in wild-type tumors (38.9% vs 16.2%, *p <*0.001) (30), with high PD-L1 expression (≥50%) observed in 17% of patients with KRAS mutation (31). We conducted an exploratory analysis to compare the efficacy of neoadjuvant immunotherapy in patients with NSCLC harboring EGFR and KRAS mutations. The results demonstrated that patients with KRAS mutations exhibited better radiological and pathological responses than those with EGFR mutations and those patients with KRAS mutations who achieved MPR also achieved pCR. In contrast, none of the patients with EGFR mutations achieved pCR. KRAS positively regulates PD-L1 expression in NSCLC, which may explain the enhanced responsiveness to immunotherapy observed in patients with KRAS mutations. Given the small numbers in these subgroups (n=11 and n=13), these observations should be validated in larger cohorts to confirm the clinical relevance. Substantial evidence indicates that tumors with EGFR mutations promote immune escape by upregulating PD-1, PD-L1, and other tumor-promoting inflammatory cytokines. Preclinical models have demonstrated that PD-L1 expression can be reduced by inhibiting EGFR in EGFR-mutated NSCLC cell lines (32–34). Additionally, the immunosuppressive TME associated with these mutations may contribute to the poor response to ICIs observed in numerous clinical trials (35–37).

Overall, this retrospective study confirmed that the response to neoadjuvant immunotherapy plus chemotherapy in patients with resectable NSCLC was not significantly affected by the presence of driver gene mutations. Additionally, this study revealed no increase in the risk of AEs or impediments to surgical resection in patients with driver gene mutations. The NeoADAURA trial demonstrated that neoadjuvant targeted therapy significantly improved the MPR rate compared to chemotherapy alone in resectable stage II–IIIB NSCLC with EGFR mutations, highlighting that the optimal neoadjuvant treatment for specific driver gene mutations remains an evolving clinical question (38).

This study has some limitations. First, it was a retrospective analysis conducted at a single institution with a limited sample size and brief follow-up period. Future studies should ideally involve larger, more diverse patient populations across multiple centers and adopt a prospective clinical trial design with longer follow-up durations. Second, another limitation of our study is the use of different types of ICIs, which may introduce confounding variables. Additionally, the relatively short follow-up period limited our survival analysis to EFS. Future studies should include extended follow-up periods to obtain OS data. This study did not further stratify specific mutation subtypes, which limited the ability to perform more detailed subgroup analyses.

In conclusion, neoadjuvant immunotherapy plus chemotherapy did not show statistically significant differences in efficacy between the mutated and wild-type groups. Neoadjuvant immunotherapy plus chemotherapy remains a promising treatment option for patients with resectable NSCLC, irrespective of genetic mutation status.

## Data Availability

The raw data supporting the conclusions of this article will be made available by the authors, without undue reservation.
