# Supplementary material for: Neoadjuvant immunotherapy plus chemotherapy for resectable non-small cell lung cancer with driver mutations: a retrospective analysis
Source: Front Immunol. 2025 Aug 27;16:1637615. doi: 10.3389/fimmu.2025.1637615 (PMC12420618; doi:10.3389/fimmu.2025.1637615)
Supplement: Supplementary file 1 [file DataSheet1.docx]

**Supplementary Material**

| **Table S1.** Characteristics of neoadjuvant immunotherapy and surgical resection | | |
| --- | --- | --- |
|  | **Mutated (*n* = 34)** | **Wild-type (*n* = 39)** |
| Type of neoadjuvant immunotherapy, *n* (%)^a^ |  |  |
| Camrelizumab | 11 (32.4) | 13 (33.3) |
| Tislelizumab | 10 (29.4) | 9 (23.1) |
| Sintilimab | 8 (23.5) | 6 (15.4) |
| Toripalimab | 2 (5.9) | 4 (10.3) |
| Nivolumab | 2 (5.9) | 2 (5.1) |
| Atezolizumab | 0 | 3 (7.7) |
| Durvalumab | 0 | 1 (2.6) |
| Pembrolizumab | 0 | 1 (2.6) |
| Adebrelimab^b^ | 1 (2.9) | 0 |
| Cycle of neoadjuvant immunotherapy, *n* (%) |  |  |
| 1^c^ | 1 (2.9) | 0 |
| 2 | 17 (50.0) | 14 (35.9) |
| 3 | 15 (44.1) | 21 (53.8) |
| 4 | 1 (2.9) | 4 (10.3) |
| Type of surgery, *n* (%) |  |  |
| Lobectomy | 29 (85.3) | 33 (84.6) |
| Sleeve lobectomy | 4 (11.8) | 5 (12.8) |
| Pneumonectomy | 1 (2.9) | 1 (2.6) |
| Completeness of resection, *n* (%) |  |  |
| R0 (negative margins in pathological examination) | 30 (88.2) | 38 (97.4) |
| R1 (positive margins in pathological examination) | 4 (11.8) | 1 (2.6) |
| Sampled lymph nodes |  |  |
| Median (IQR) | 21 (18–24) | 21 (17–24) |
| Data are presented as number (%) or median (IQR).  IQR, interquartile range.  Percentages may not total 100 due to rounding.  ^a^ All patients were treated with immunotherapy-based neoadjuvant therapy plus either albumin-bound paclitaxel and platinum-based chemotherapy (for participants with squamous histologic features) or pemetrexed and platinum-based chemotherapy (for those with non-squamous histologic features).  ^b^ One patient was enrolled in a randomized, double-blind, multicenter, phase IB/III trial of SHR-1316 (adebrelimab) or placebo plus chemotherapy in the perioperative treatment of resectable stage II or III non-small cell lung cancer (protocol no. SHR-1316-III-303, V3.0/2021-07-20).  ^c^ One patient received one cycle of sintilimab plus pemetrexed and platinum-based chemotherapy and stopped neoadjuvant immunotherapy due to a reduction in the platelet count. Finally, surgical resection was promptly performed on the patient when the criteria for treatment resumption was satisfied. | | |

| **Table S2.** Treatment-related Adverse Events during Neoadjuvant Immunotherapy. | | | | | | |
| --- | --- | --- | --- | --- | --- | --- |
| Events: no. (%) | Mutated (n=34) | | | Wild-type (n=39) | | |
|  | Any Grade | Grade 2 | Grade 3 | Any Grade | Grade 2 | Grade 3 |
| Anemia | 27(79.4) | 2(5.9) | 0 | 25(64.1) | 8(20.5) | 1(2.6) |
| Nausea | 17(50.0) | 7(20.6) | 2(5.9) | 15(38.5) | 7(17.9) | 1(2.6) |
| Fatigue | 13(38.2) | 6(17.6) | 1(2.9) | 17(43.6) | 3(7.7) | 2(5.1) |
| Vomiting | 13(38.2) | 4(11.8) | 1(2.9) | 11(28.2) | 3(7.7) | 1(2.6) |
| Neutrophil count decreased | 9(26.5) | 5(14.7) | 0 | 13(33.3) | 5(12.8) | 4(10.3) |
| White cell count decreased | 8(23.5) | 4(11.8) | 0 | 13(33.3) | 7(17.9) | 1(2.6) |
| Alanine aminotransferase increased | 12(35.3) | 2(5.9) | 1(2.9) | 8(20.5) | 2(5.1) | 0 |
| Increased serum thyroid-stimulating hormone | 4(11.8) | 3(8.8) | 0 | 6(15.4) | 2(5.1) | 0 |
| Decreased serum thyroid-stimulating hormone | 5(14.7) | 1(2.9) | 0 | 7(17.9) | 2(5.1) | 0 |
| Constipation | 5(14.7) | 5(14.7) | 0 | 6(15.4) | 1(2.6) | 1(2.6) |
| Platelet count decreased | 4(11.8) | 2(5.9) | 1(2.9) | 4(10.3) | 0 | 0 |
| Blood creatinine levels increased | 2(5.9) | 0 | 0 | 2(5.1) | 0 | 0 |
| Pneumonitis | 1(2.9) | 0 | 1(2.9) | 1(2.6) | 1(2.6) | 0 |
| Diarrhea | 1(2.9) | 1(2.9) | 0 | 1(2.6) | 1(2.6) | 0 |
| Skin disorders (rash) | 0 | 0 | 0 | 3(7.7) | 0 | 1(2.6) |
| Data are n (%). No grade 4 or 5 adverse events were reported. | | | | | | |


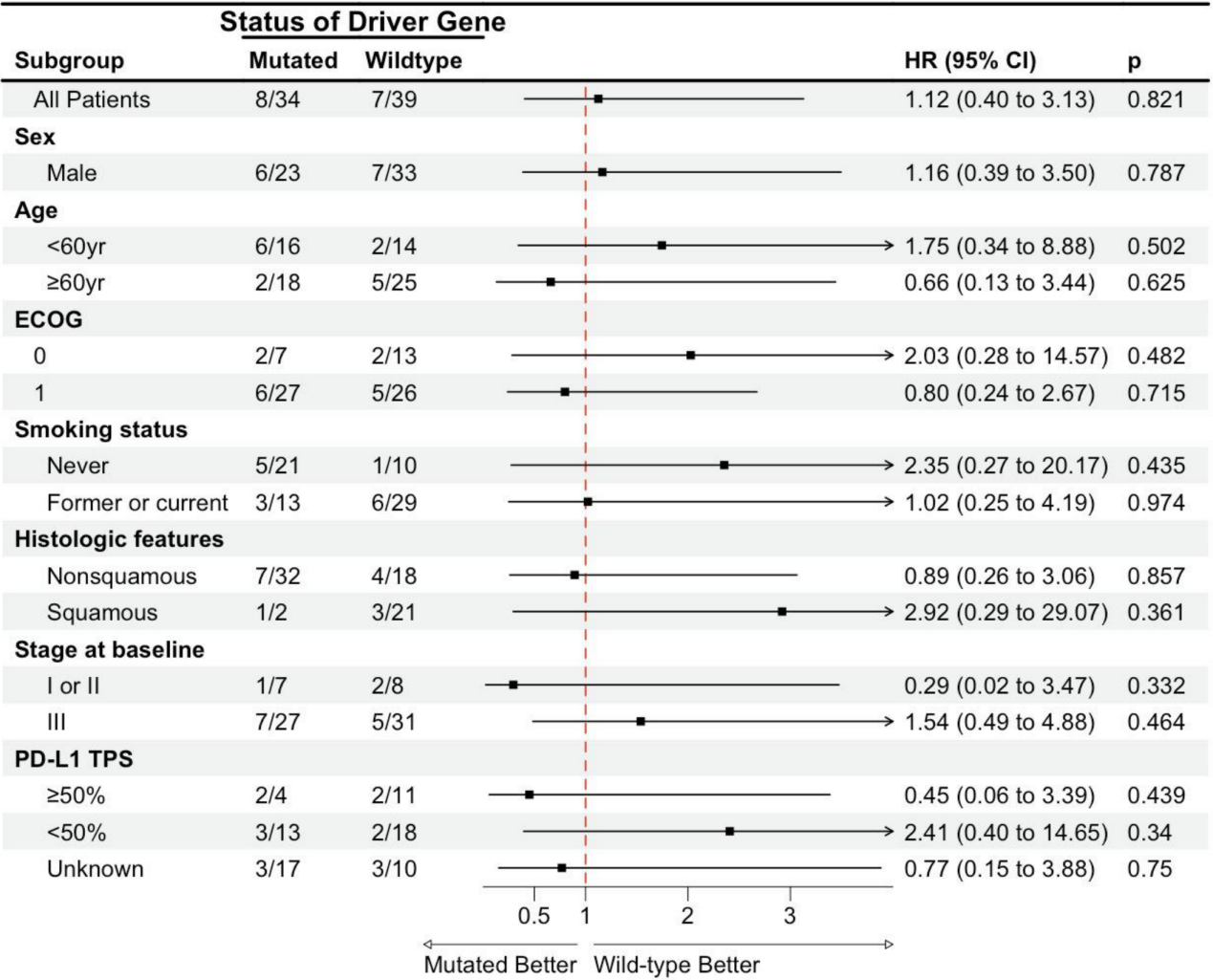


Figure S1. Forest plot of event-free survival.

ECOG, Eastern Cooperative Oncology Group; TPS, tumor proportion score; PD-L1, programmed death ligand 1.
